# Histone H3 Acetylation Is Involved in Retinoid Acid-Induced Neural Differentiation through Increasing Mitochondrial Function

**DOI:** 10.3390/biomedicines11123251

**Published:** 2023-12-08

**Authors:** Yang Zhang, Xinjuan Wang, Qing Mu, Xueyu Hou, Weidong Yu, Jingzhu Guo

**Affiliations:** 1 Department of Pediatric, Peking University People’s Hospital, Beijing 100044, China; 2 Department of Central Laboratory and Institute of Clinical Molecular Biology, Peking University People’s Hospital, Beijing 100044, China

**Keywords:** retinoid acid, neural differentiation, mitochondrial function, histone acetylation, histone acetyltransferase

## Abstract

Histone acetylation and mitochondrial function contribute importantly to neural differentiation, which is critically associated with neurodevelopmental disorders such as Down Syndrome (DS). However, whether and how histone acetylation regulates mitochondrial function and further affects neural differentiation has not been well described. In this study, when treated with retinoid acid (RA), the *human* neuroblastoma SH-SY5Y cell line was used as a neural differentiation model. We found that the acetylation of histone H3, especially H3 lysine 14 acetylation (H3K14ac), and mitochondrial function, including biogenesis and electron transport chain, were enhanced during neural differentiation. Specific inhibition of histone acetyltransferases (HATs) induced neural differentiation deficits, accompanied by downregulation of mitochondrial function. Furthermore, RA receptors (RARs) interacting with HATs were involved in the increased H3K14ac and the enhanced mitochondrial function during the neural differentiation process. Finally, receptor-interacting protein 140 (RIP140), a co-repressor of RARs, was also involved in regulating histone acetylation. RIP140 overexpression inhibited histone acetylation and mediated negative feedback on target genes which are involved in RA signaling. These findings evidenced that when interacting with RARs which had been negatively regulated by RIP140, RA promoted neural differentiation by promoting H3K14ac and enhanced mitochondrial function. This provides a molecular foundation for further investigations into abnormal neural development.

## 1. Introduction

Neural differentiation is an essential process in central nervous system (CNS) development [1]. Deficits in neural differentiation lead to significantly delayed brain development and impaired cognitive ability, which is seem in disorders such as Down Syndrome (DS) [2]. Therefore, a better understanding of how neural differentiation is regulated and maintained will expand the strategies for treating neurodevelopmental disorders.

Chromatin modifications, particularly histone acetylation, have critical functions in neural differentiation and have been increasingly appreciated in early neurodevelopment. Acetylation in the N-terminal tails of histone H3 promote neural differentiation by regulating gene expression in a spatial and temporal manner [3,4]. Moreover, histone H3 lysine 14 acetylation (H3K14ac) is required during melatonin-induced neural stem cell (NSC) differentiation [5]. Increasing histone acetylation results in enhanced neural differentiation and hippocampal neurogenesis [4,6]. Acetylation homeostasis is maintained by histone acetyltransferases (HATs) and histone deacetylases (HDACs), which transfer acetyl groups to or remove acetyl groups from histone residues, altering chromatin structure and regulating gene transcription [7]. It is critical for HATs, such as CREB-binding protein (CBP), E1A-binding protein P300 (P300), and P300/CBP-associated factor (PCAF), to promote memory through inducing memory-related gene transcription [8]. Enhanced histone acetylation by HDAC inhibitors has been shown to be protective against the development of neurodegenerative disease [9]. However, the mechanism underlying how histone acetylation regulates the process of neural differentiation has not been well described.

In our and other previous work, an association between mitochondrial function and neural differentiation was observed. We previously demonstrated that peroxisome proliferator-activated receptor-γ coactivator-1α (PGC-1α), a key modulator of mitochondrial biogenesis, and several nuclear-encoded mitochondrial genes (NEMGs) were significantly upregulated during vitamin A-induced neural differentiation [10]. Moreover, the effect of melatonin on NSC differentiation also depends on modulating mitochondrial activity [11]. Mitochondrial complex I function has been shown to be essential for neural stem/progenitor cell differentiation [12]. It is well known that mitochondria are important for promoting energy transport in neurons and controlling fundamental processes in neuroplasticity [13]. Disturbances to mitochondrial function are closely associated with the pathogenesis of DS [14]. Due to the direct link between mitochondria and neural differentiation, we asked whether histone acetylation promotes neural differentiation by regulating mitochondrial function, such as biogenesis and mitochondrial complex activity.

In the present study, the SH-SY5Y cell line induced by retinoid acid (RA) was used to evaluate changes in histone acetylation during neural differentiation. We found RA activates HATs and enhances histone H3 acetylation in SH-SY5Y cells. Histone acetylation was involved in regulating mitochondrial morphology and function, thereby affecting the neural differentiation of SH-SY5Y cells. Mechanistically, RA-induced changes in histone acetylation were regulated by retinoid acid receptor (RAR) and its co-repressor receptor-interacting protein 140 (RIP140). Collectively, we revealed an epigenetic mechanism coupling mitochondrial function to neural differentiation and provided novel insight into therapies for neurodevelopmental disorders.

## 2. Materials and Methods

### 2.1. Cell Culture

We obtained the SH-SY5Y cell line from the Chinese Academy of Sciences (Shanghai, China). MEM/F12 (1:1) medium (HyClone, Logan, UT, USA) supplemented with 10% FBS, 1% nonessential amino acids (NEAAs), 1% sodium pyruvate, and 1% penicillin/streptomycin (Gibco, Waltham, MA, USA) was used to culture the SH-SY5Y cells. N2a cells (a mouse neuroblastoma cell line) were obtained from the National Platform of Experimental Cell Resource for Sci-Tech (Beijing, China). DMEM supplemented with 10% FBS and 1% penicillin/streptomycin (Gibco, USA) was used to culture the N2a cells. The cells were incubated in a humidified atmosphere of 5% CO_2_ at 37 °C.

To induce neural differentiation, SH-SY5Y cells were exposed to 1 µM RA (MCE, Shanghai, China) for 1, 3, 5, and 7 days and N2a cells were treated with 10 µM RA for 3 days [10]. To identify the roles of key enzymes and receptors during neural differentiation, the HAT activity inhibitor C646 (Sigma, St. Louis, MO, USA), RARα antagonist BMS195614, and RARβ antagonist LE135 (Glpbio, Montclair, CA, USA) were supplemented during the RA treatment. The final concentrations of C646, BMS195614, and LE135 were 15 μM, 10 μM, and 10 μM, respectively [5,10].

### 2.2. Generation of a Stable RIP140-Overexpressing Cell Line

Plasmid constructs were designed as previously described [15]. To generate a stable cell line overexpressing the *RIP140* gene, 2 × 10^5^ N2a cells were transfected with 2 µg pCMV6-*RIP140*-GFP vectors (Origene, Rockville, MD, USA) using Lipofectamine^TM^ 2000 (Invitrogen, Carlsbad, CA, USA). After transfection for 48 h, puromycin (2 µg/mL) was used to select transfected cells. The antibiotic culture medium was replenished every 48 h and antibiotic selection was maintained for 15 to 20 days. The mRNA and protein expression of RIP140 was used to identify the positive clones.

### 2.3. Hematoxylin and Eosin Staining (H&E Staining)

Cells were washed three times with phosphate-buffered saline (PBS) and then fixed with 4% paraformaldehyde (PFA) at room temperature (RT) for 15 min. After being permeabilized with 0.2% Triton X-100 at RT for 30 min, the cells were stained with hematoxylin (Solarbio, Beijing, China) for 1 min and then stained with eosin (Solarbio, China) for 4 min. The neural differentiation rate was calculated according to the Sandquist method [10]. Cells with more than one protuberance longer than the diameter of the cell body were considered differentiated cells. The differentiation rate was defined as the average percentage of differentiated cells in three randomly selected fields in each group.

### 2.4. Immunofluorescence

Cells were washed three times and fixed with 4% PFA at RT for 15 min. The fixed cells were then permeabilized with 0.2% Triton X-100 at RT for 30 min. The pretreated cells were blocked with 10% goat serum for 30 min before incubation with anti-Tuj1 antibody (1:200; Proteintech, Wuhan, China), anti-MAP2 antibody (1:100; Beyotime, Shanghai, China), anti-acetyl-histone H3 antibody (1:500; Millipore, Burlington, MA, USA), and anti-PGC1-α antibody (1:200; Proteintech, China) at 4 °C overnight. The cells were then incubated with secondary antibodies conjugated to FITC or CY3 (1:200; Servicebio, Wuhan, China) away from light for 1 h. Cell nuclei were counterstained with DAPI (Sigma, USA) for 10 min. Finally, the sample was randomly photographed, and the fluorescence intensity was analyzed from three different fields of view in each group from three independent experiments using ImageJ software (version 2.0.0-rc-69).

### 2.5. Transmission Electron Microscopy (TEM)

Cells were harvested and fixed with 2.5% glutaraldehyde (Solarbio, China) at 4 °C. The cells were then fixed in 1% OsO_4_ at 4 °C for 1 h, washed three times with PBS, and dehydrated in ascending grades of ethanol (50%, 70%, 90%) at 4 °C for 10 min each, followed by three rounds of dehydration in 100% ethanol at RT for 10 min. Subsequently, the specimens were transferred to an alcohol and propylene oxide (1:1) mixture and then pure propylene oxide at RT for 10 min. All samples were embedded in Epon 812 (EMS, China) and sliced using an ultramicrotome (Leica Ultracut UCT/UC6, Wetzlar, Germany). After staining with lead and uranium, the samples were observed using transmission electron microscopy (TEM) (FEI Tecnai Spirit, Hillsboro, OR, USA) [16].

### 2.6. Detection of Reactive Oxygen Species (ROS)

Treated cells were incubated with DCFH-DA (Beyotime, China) at a concentration of 10 µmol/L for 30 min at 37 °C to detect intracellular ROS levels. Fluorescence was recorded at 488 nm using fluorescence microscopy (Leica). The percentage of cells positive for the probe was detected through flow cytometry (BD FACSCelesta, BD Bioscience, Franklin Lakes, NJ, USA) and analyzed using FlowJo software (version 10) [17].

### 2.7. RNA Preparation and Real-Time Quantitative PCR (RT-qPCR)

The total RNA of cells was extracted using a total RNA isolation system (Omega, USA) and the Revert Aid First Strand cDNA Synthesis Kit (Thermo Scientific, Waltham, MA, USA) was then used to perform reverse transcription. RT-qPCR was performed using the SYBR Green Real-time PCR Master Mix (TOYOBO, Osaka, Japan) and an RT–PCR instrument (Bio-Rad, Hercules, CA, USA). The results were calculated by normalizing the expression of target genes to that of GAPDH or β-actin using the delta C_t_ method. The primers for the genes were designed using Primer 5 software and are listed in Table 1.

### 2.8. Protein Preparation and Western Blot

The harvested cells were lysed using RIPA buffer containing protease inhibitor and PMSF (Solarbio, China) for 20 min at 4 °C. Then lysates were then centrifuged for 15 min at 4 °C and the total protein was obtained. Histone extraction was performed using the EpiQuik Total Histone Extraction Kit (Epigentek, Farmingdale, NY, USA). The BCA Protein Assay Kit (Thermo Scientific, USA) was then used to measure the concentration of the protein samples. Equivalent amounts of protein from each group were loaded onto a 6–15% gel for SDS-PAGE (Solarbio, China). The proteins were then transferred to a 0.45 µm PVDF membrane (Millipore, USA) in transfer buffer. The membrane was blocked with 5% milk in TBST for 1 h at room temperature and probed with the following antibodies at 4 °C overnight: anti-Tuj1 antibody (1:5000; Proteintech, China), anti-MAP2 antibody (1:1000; Beyotime, China), anti-RIP140 antibody (1:500; Sigma, USA/1:1000; ABclonal, Wuhan, China), anti-PGC1-α antibody (1:5000; Proteintech, China), anti-acetyl-H3 antibody (1:10,000; Millipore, USA), anti-acetyl-H3 (lys14) antibody (1:1000; CST, Moorestown, NJ, USA), anti-PCAF antibody (1:1000; CST, USA), anti-CBP antibody (1:1000; CST, USA), anti-P300 antibody (1:1000; CST, USA), and anti-RARα antibody (1:1000; Proteintech, China). HRP-linked secondary antibodies (1:2000; CST, USA) were then applied at RT for 1 h the following day. Finally, an ultrasensitive chemiluminescence kit (Beyotime, China) was used to visualize the bands in the ECL Imaging System (Bio-Rad, USA).

### 2.9. Co-Immunoprecipitation

For the immunoprecipitation analysis, the protein extracts were incubated with 1 µg anti-RARα antibody (Proteintech, China) or control IgG antibody (Beyotime, China) overnight at 4 °C. The complexes were then incubated with protein-A/G agarose beads (Santa Cruz, Santa Cruz, CA, USA) for 6 h at 4 °C the next day. The immunoprecipitates were washed four times and then boiled for subsequent Western blotting.

### 2.10. HAT Activity Assay

The nuclear extracts isolated using the EpiQuik Nuclear Extract Kit (Epigentek, USA) were prepared for the HAT activity assay. The HAT activity was then measured using the EpiQuik HAT Activity/inhibition Assay Kit (Epigentek, USA) according to the manufacturer’s instructions. The results were analyzed using a microplate reader at an absorbance of 440 nm [18].

### 2.11. Statistical Analysis

Statistical analysis was performed using GraphPad Prism 8.4.0 software. Data from three independent repeats were expressed as the mean ± standard error of the mean (SEM). Student’s *t*-test or one-way analysis of variance (ANOVA) were used to calculate statistical significance. *p* values less than 0.05 were considered statistically significant.

## 3. Results

### 3.1. The Neural Differentiation Model Is Established with SH-SY5Y Cells Induced by RA

**Figure 1:**
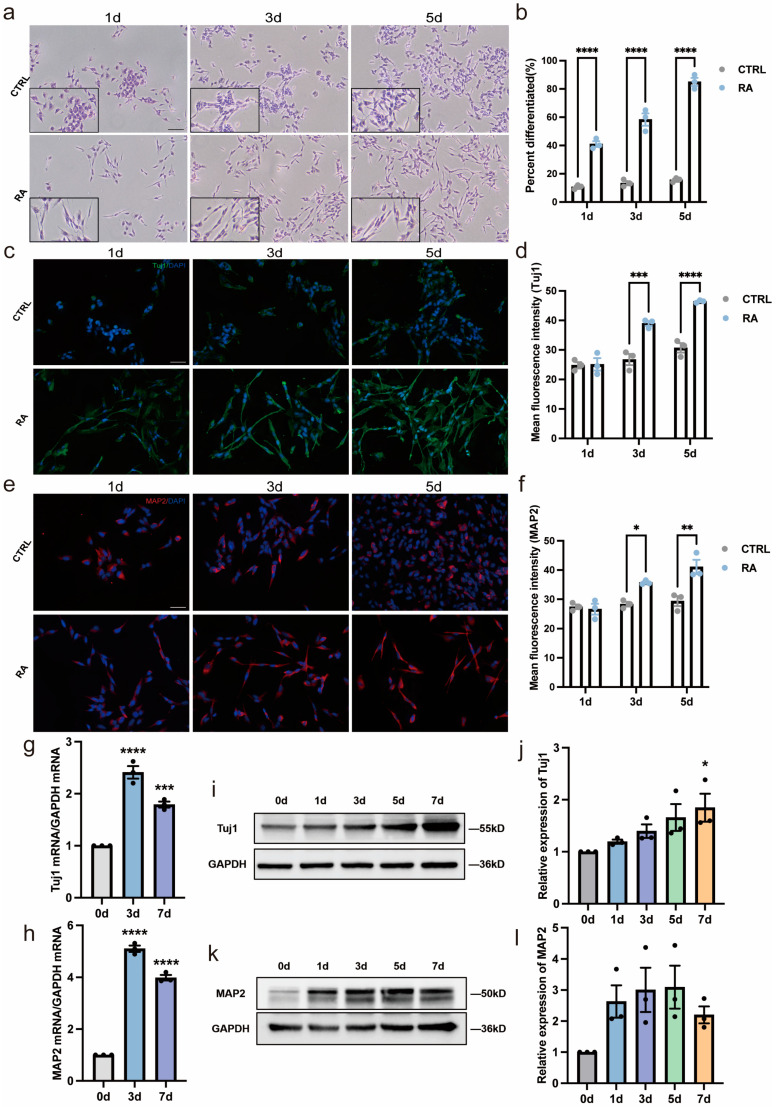
Establishment of neural differentiation model using RA treatment of SH-SY5Y cells. (**a**) Representative H&E staining images of cell morphology of SH-SY5Y after RA treatment for 1, 3, and 5 days. Scale bar, 100 µm. Images in the black box show cells at a higher magnification. (**b**) RA treatment advances differentiation of SH-SY5Y in a time-dependent manner. The differentiation rate was defined as the average percentage of differentiated cells in three fields. **** *p* < 0.0001; two-way ANOVA. (**c**–**f**) Expression of neuronal differentiation markers Tuj1 and MAP2 in SH-SY5Y after RA treatment for 1, 3, and 5 days. Cells are labeled with Tuj1 (green) and MAP2 (red), and nuclei were counterstained with DAPI (blue). Scale bar, 50 µm. * *p* < 0.05, ** *p* < 0.01, *** *p* < 0.001, **** *p* < 0.0001; two-way ANOVA. (**g**,**h**) RA treatment increases *Tuj1* and *MAP2* expression at mRNA level. *n* = 3; *** *p* < 0.001, **** *p* < 0.0001; one-way ANOVA. (**i**–**l**) Relative protein expression of Tuj1 and MAP2 increased after RA treatment. Western blot analysis of Tuj1 and MAP2 is shown above the corresponding histogram. *n* = 3; * *p* < 0.05; one-way ANOVA.

The RA-induced SH-SY5Y cell line is a widely applied model for neural differentiation research [19]. H&E staining showed that RA promoted neuron-like morphological changes in SH-SY5Y cells (Figure 1a). According to the Sandquist method, differentiated cells were characterized by extending neurites equal to or longer than the diameter of the soma. After 1 μM RA treatment for 1, 3, and 5 days, the rates of neural differentiation were 41%, 58.33%, and 85%, respectively, while the differentiation rates in the control groups were 10.67%, 13.33%, and 15.67%, respectively (Figure 1b). Immunofluorescent staining showed that the neural differentiation markers neuron-specific class III beta-tubulin (Tuj1) and microtubule-associated protein 2 (MAP2) were increased in SH-SY5Y cells after exposure to RA (Figure 1c–f). The mRNA levels of both neuronal markers were upregulated after RA treatment (Figure 1g,h) and increased protein expression of Tuj1 and an upward tendency of MAP2 expression were also observed using Western blot analysis (Figure 1i–l). In summary, based on the changes in morphology and the expression of neural differentiation markers, our in vitro RA-induced neural differentiation model was established.

### 3.2. RA Activates PCAF/CBP/P300 and Increases the Acetylation of Lysine 14 in Histone H3 during Neural Differentiation

**Figure 2:**
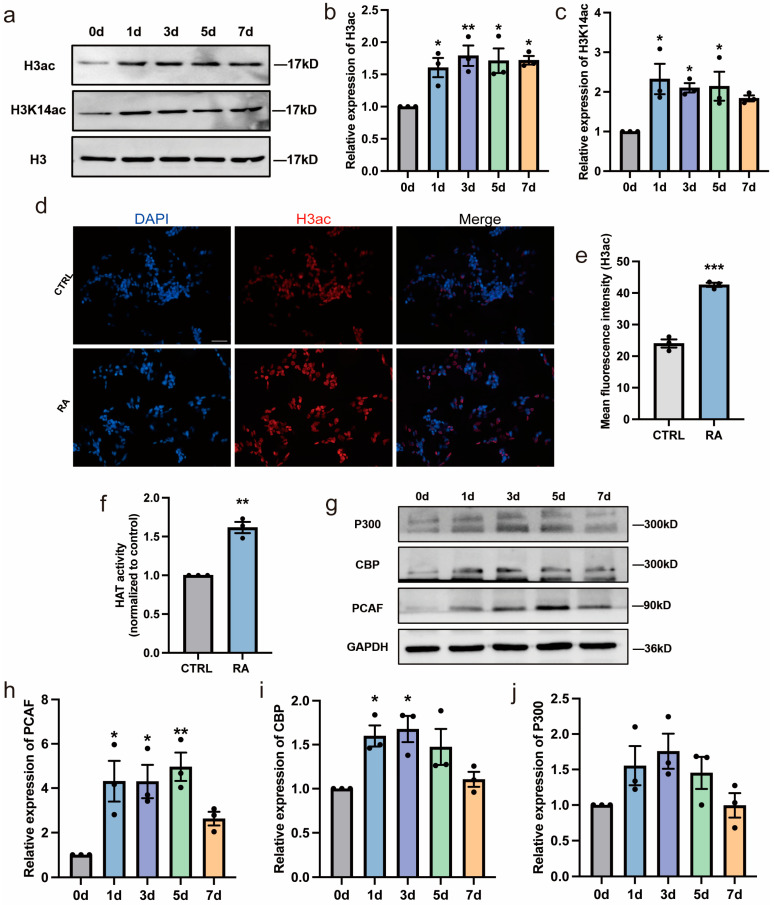
RA increases the acetylation of histone H3 during neural differentiation of SH-SY5Y cells. (**a**–**c**) Western blot analysis of H3K14ac and H3ac in SH-SY5Y cells exposed to RA for 1, 3, 5, and 7 days. *n* = 3; * *p* < 0.05, ** *p* < 0.01; one-way ANOVA. (**d**,**e**) SH-SY5Y cells with or without RA treatment for 3 days were immunostained with anti-H3ac (red) and nuclei were counterstained with DAPI (blue). Scale bar, 50 µm. *** *p* < 0.001; *t*-test. (**f**) SH-SY5Y cells with or without RA treatment for 3 days were subjected to a HAT activity assay. *n* = 3; ** *p* < 0.01; *t*-test. (**g**–**j**) Western blot analysis of relative protein expression of HATs (PCAF, CBP, and P300) in SH-SY5Y cells after RA treatment for 1, 3, 5, and 7 days. *n* = 3; * *p* < 0.05, ** *p* < 0.01; one-way ANOVA.

Histone acetylation has been shown to play critical roles during neural differentiation [3,20] and RA has been reported to be associated with histone acetylation [21,22]. To investigate whether histone acetylation is involved in regulating RA-induced neural differentiation of SH-SY5Y cells, changes in histone H3 acetylation were evaluated in the present study. Acetylated histone H3 from RA-treated and control cells was detected using specific antibodies and Western blot analysis. The total acetylation of histone H3 (H3ac) and acetylation of histone H3 lysine 14 (H3K14ac) levels in SH-SY5Y cells increased significantly after RA treatment (Figure 2a–c). The immunofluorescence staining also showed an increase in H3ac in the RA-treated cells versus the control cells (Figure 2d,e). HATs directly regulate the histone acetylation status by transferring acetyl groups to lysine residues of histone tails. Therefore, the activity and protein expression of several major HATs, including PCAF, CBP, and P300, were examined. A significant increase in HAT activity was detected using the HAT assay (Figure 2f). Moreover, Western blot analysis showed that RA promoted the expression of PCAF and CBP proteins. There was also an upward trend in the expression of P300 (Figure 2g–j). These results suggest that RA activates HATs and increases the acetylation of histone H3 during the neural differentiation of SH-SY5Y cells.

### 3.3. RA Increases Mitochondrial Biogenesis and Function during Neural Differentiation

**Figure 3:**
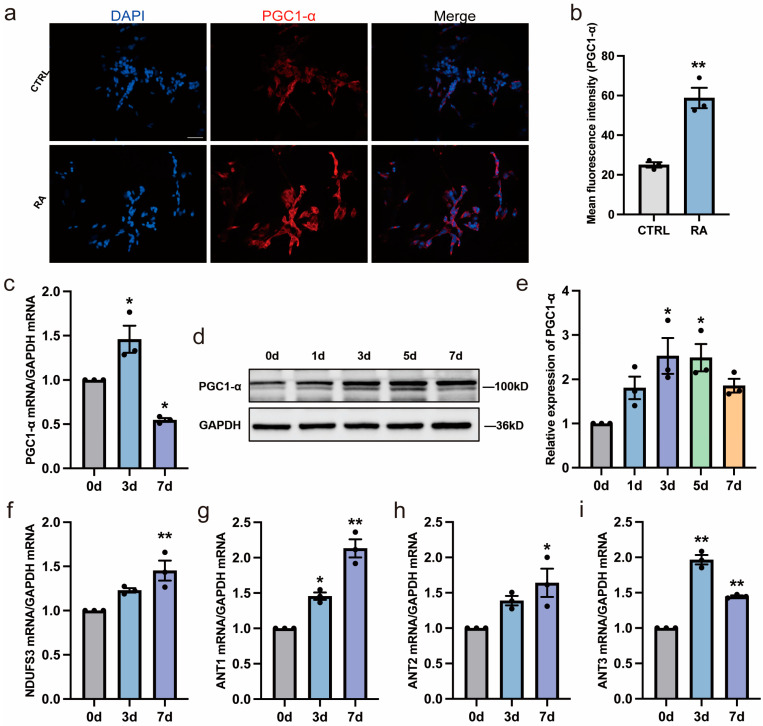
RA increases mitochondrial biogenesis and function during neural differentiation of SH-SY5Y cells. (**a**,**b**) SH-SY5Y cells with or without RA treatment for 3 days were immunostained with anti-PGC-1α (red) and nuclei were counterstained with DAPI (blue). Scale bar, 50 µm. ** *p* < 0.01; *t*-test. (**c**) RT-qPCR analysis of relative *PGC-1α* mRNA expression in SH-SY5Y cells after RA treatment for 3 and 7 days. *n* = 3; * *p* < 0.05; one-way ANOVA. (**d**,**e**) Western blot analysis of relative PGC-1α protein expression in SH-SY5Y cells after RA treatment for 1, 3, 5, and 7 days. *n* = 3; * *p* < 0.05; one-way ANOVA. (**f**–**i**) RT-qPCR analysis of relative mRNA expression of mitochondrial function-related genes (*NDUFS3*, *ANT1*, *ANT2*, *ANT3*) in SH-SY5Y cells after RA treatment for 3 and 7 days. *n* = 3; * *p* < 0.05, ** *p* < 0.01; one-way ANOVA.

In our previous work, mitochondrial function was shown to be closely associated with the process of neural differentiation [10]. As shown in Figure 3a,b, PGC-1α, a key regulator of mitochondrial biogenesis activity, increased after RA treatment with immunofluorescent staining. Further, the PGC-1α expression in mRNA and protein level were detected in the third and seventh day with RA treatment. It increased initially and then decreased by 7 days (Figure 3c–e). The decreased expression of PGC-1α may be regulated simultaneously by other inhibitors, such as RIP140 [23]. In addition, several mitochondrial function-related genes (NEMGs), including *NDUFS3*, *ANT1*, *ANT2*, and *ANT3*, were upregulated after exposure to RA, indicating enhanced mitochondrial complex function during RA-induced neural differentiation (Figure 3f–i).

### 3.4. HAT Activity Inhibitor Inhibits Neural Differentiation of SH-SY5Y Cells

**Figure 4:**
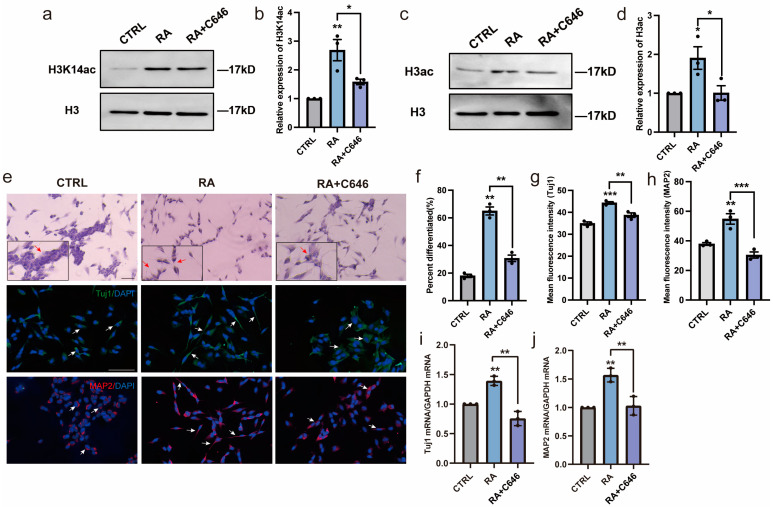
Histone acetylation is important for RA’s ability to promote neural differentiation of SH-SY5Y cells. SH-SY5Y cells were treated with DMSO or RA or pretreated with C646 for 3 h followed by RA for 3 days. (**a**–**d**) Western blot analysis of H3K14ac and H3ac in the experimental groups. *n* = 3; * *p* < 0.05, ** *p* < 0.01; one-way ANOVA. (**e**) Changes in morphology and expression of neuronal differentiation markers Tuj1 (green) and MAP2 (red) in SH-SY5Y cells. Scale bar, 50 µm. Images in the black box show cells at a higher magnification. Red or white arrows show representative changes in morphology. (**f**–**h**) Differentiation rates and mean fluorescence intensity of Tuj1 and MAP2. *n* = 3; ** *p* < 0.01, *** *p* < 0.001; one-way ANOVA. (**i**,**j**) RT-qPCR analysis of relative mRNA expression of the neural differentiation markers *Tuj1* and *MAP2*. *n* = 3; ** *p* < 0.01; one-way ANOVA.

To further investigate whether histone acetylation plays an important role in RA-induced neural differentiation, C646, a specific inhibitor of HAT CBP/P300 activity, was used to treat SH-SY5Y cells simultaneously with RA. H3ac and H3K14ac were significantly decreased after inhibition of HAT activity with C646 treatment for 3 days (Figure 4a–d). Interestingly, the increased neural differentiation rates caused by RA stimulation were almost blocked when H3ac and H3K14ac were inhibited by C646 (Figure 4e,f). This suggested that histone acetylation was important for maintaining normal neurite outgrowth. Furthermore, the increased expression of the neural differentiation markers Tuj1 and MAP2 induced by RA stimulation were subsequently blocked (Figure 4e,g–j), indicating that histone acetylation plays a crucial role in RA-induced neural differentiation and that RA promotes neural differentiation by activating HATs.

### 3.5. Histone Acetylation Is Involved in Regulating Mitochondrial Morphology and Function 

**Figure 5:**
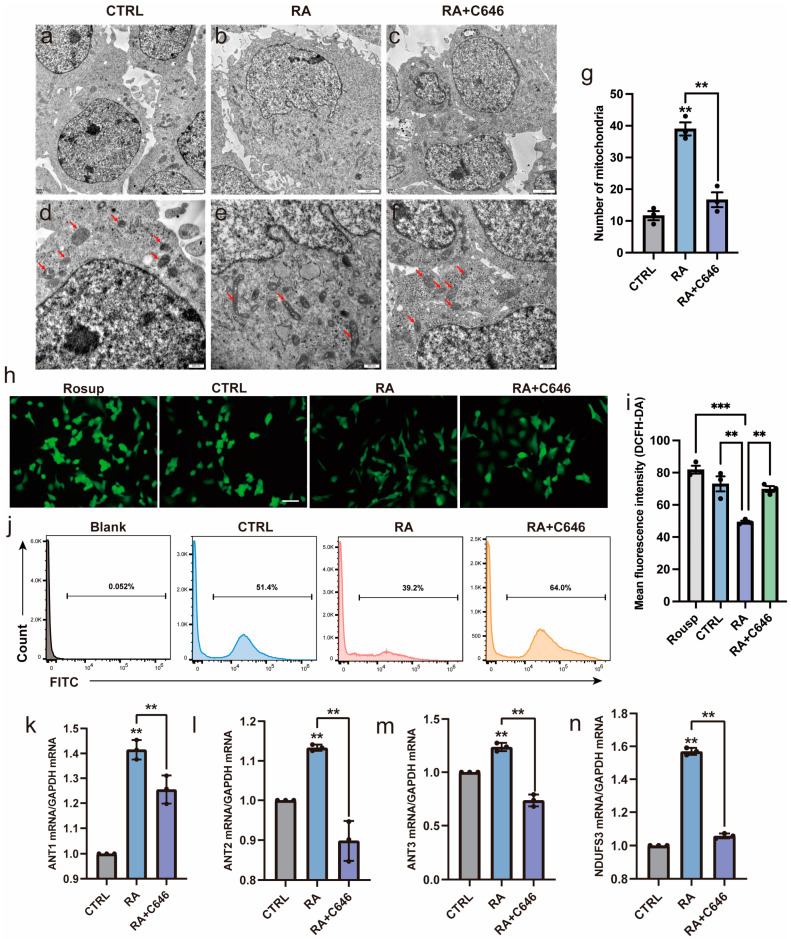
Histone acetylation is involved in regulating mitochondrial morphology and function. SH-SY5Y cells were treated with DMSO or RA or pretreated with C646 for 3 h followed by RA for 3 days. (**a**–**c**) Ultrastructural images of SH-SY5Y cells. RA induced an irregular morphology in the cells with microvillus projections, lowered nuclear–cytoplasmic ratios, and increased the number of mitochondria, and C646 blocked these changes. Scale bar, 2 µm. (**d**–**f**) Ultrastructural images of mitochondria. RA induced the mitochondria in SH-SY5Y cells to change from a spherical shape with immature cristae to a long strip shape with well-developed cristae, and C646 suppressed these changes. Red arrows show the mitochondria. Scale bar, 500 nm. (**g**) Number of mitochondria in SH-SY5Y cells. The mitochondrial number was calculated as the average of three fields and there was at least one complete cell in each field. ** *p* < 0.01; one-way ANOVA. (**h**–**j**) Intracellular ROS generation in SH-SY5Y cells was assessed using the ROS-sensitive fluorometric probe DCFH-DA, fluorescence microscopy, and flow cytometry. Cells treated with Rosup were the positive control. Rosup, a compound mixture, can strongly induce the generation of ROS. Scale bar, 100 µm. ** *p* < 0.01, *** *p* < 0.001; one-way ANOVA. (**k**–**n**) RT-qPCR analysis of relative mRNA expression of mitochondrial function-related genes (*NDUFS3*, *ANT1*, *ANT2*, *ANT3*). *n* = 3; ** *p* < 0.01; one-way ANOVA.

Whether histone acetylation is involved in regulating mitochondrial morphology and function during neural differentiation was also investigated. As illustrated in Figure 5, when C646 inhibited histone acetylation, the RA-induced changes in mitochondrial morphology and function were blocked. The cellular ultrastructure of SH-SY5Y cells was observed using TEM. After RA treatment, SH-SY5Y cells changed from a regular oval shape to an irregular shape with microvillous protrusions, the nuclear–cytoplasmic ratio decreased, and the number of mitochondria increased. Further observations at a high magnification showed that the mitochondria changed from spherical to club-shaped and that the mitochondrial crest was mature with a clear structure (Figure 5a–g). Moreover, previous studies have demonstrated that neural differentiation requires a metabolic shift towards oxidative phosphorylation and correlates with a functional increase in the ROS scavenging mechanisms [24]. Here, we found that intracellular ROS levels were decreased after RA treatment and all the changes caused by RA stimulation were inhibited after adding C646 (Figure 5h–j), which was consistent with previous studies. In addition, mitochondrial function-related genes were downregulated after C646 treatment (Figure 5k–n).

### 3.6. RA Promotes HATs and Increases Histone Acetylation via RA Signaling during Neural Differentiation

**Figure 6:**
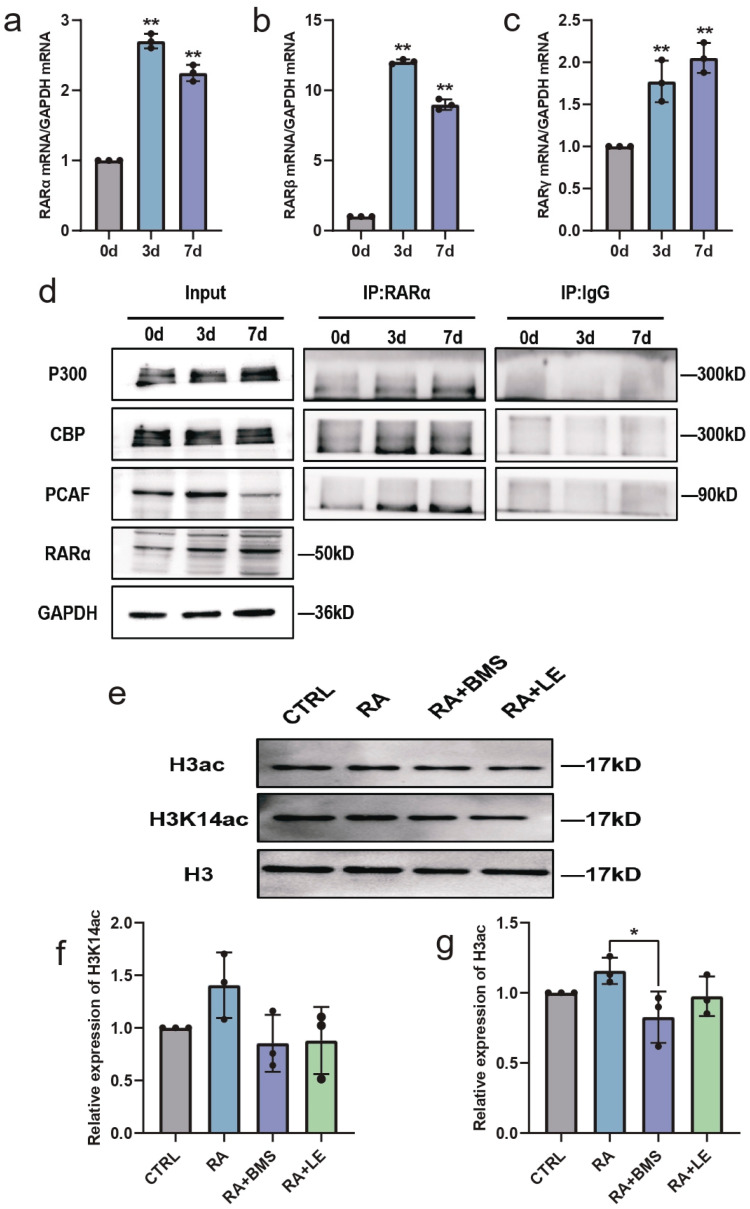
RA promotes histone acetylation through RA signaling during neural differentiation of SH-SY5Y cells. (**a**–**c**) RT-qPCR analysis of relative mRNA expression of *RARα*, *RARβ*, and *RARγ* in SH-SY5Y cells after RA treatment for 3 and 7 days. *n* = 3; ** *p* < 0.01; one-way ANOVA. (**d**) Co-immunoprecipitation was performed in SH-SY5Y cells cultured in maintenance medium or stimulated with RA for 3 and 7 days. Immunoprecipitation was conducted using anti-RARα followed by Western blot analysis of PCAF, CBP, and P300. (**e**–**g**) Western blot analysis of H3K14ac and H3ac in SH-SY5Y cells treated with DMSO or RA or pretreated with BMS195614/LE135 for 24 h followed by RA for 3 days. *n* = 3; * *p* < 0.05; one-way ANOVA.

**Figure 7:**
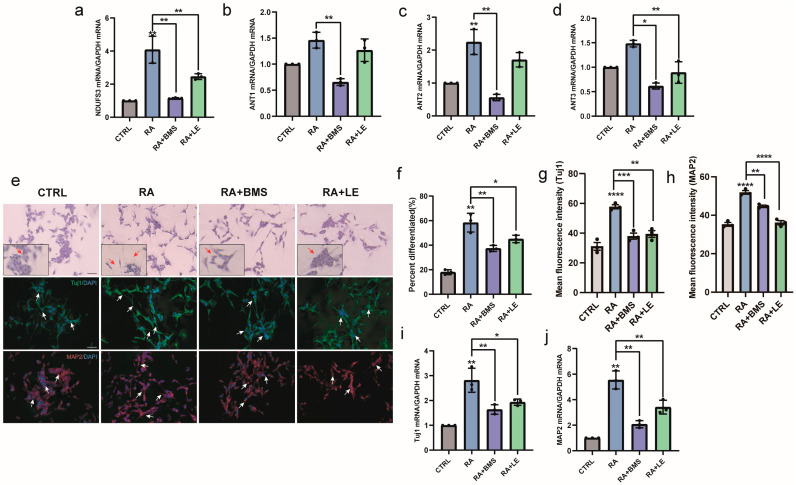
Inhibition of RA signaling downregulates mitochondrial function and blocks neural differentiation. SH-SY5Y cells were treated with DMSO or RA or pretreated with BMS195614/LE135 for 24 h followed by RA for 3 days. (**a**–**d**) RT-qPCR analysis of relative mRNA expression of mitochondrial function-related genes (*NDUFS3*, *ANT1*, *ANT2*, *ANT3*). *n* = 3; * *p* < 0.05, ** *p* < 0.01; one-way ANOVA. (**e**) Changes in morphology and expression of neuronal differentiation markers Tuj1 (green) and MAP2 (red). Scale bar, 50 µm. Images in the black box show cells at a higher magnification. Red or white arrows show representative changes in morphology. (**f**–**h**) Differentiation rates and mean fluorescence intensity of Tuj1 and MAP2. *n* = 3; * *p* < 0.05, ** *p* < 0.01, *** *p* < 0.001, **** *p* < 0.0001; one-way ANOVA. (**i**,**j**) RT-qPCR analysis of relative mRNA expression of the neural differentiation markers *Tuj1* and *MAP2*. *n* = 3; * *p* < 0.05, ** *p* < 0.01; one-way ANOVA.

RA performs most of its physiological functions by activating three RAR subtypes, RARα, RARβ, and RARγ, and triggers the RA signaling pathway [25]. RARs are widely expressed in the brain and are considered key regulators of neural differentiation [26]. During neural differentiation of RA-induced SH-SY5Y cells, the mRNA expression of *RARα*, *RARβ*, and *RARγ* was upregulated (Figure 6a–c). To identify how RA promotes HAT expression, we investigated whether RARs could recruit HATs during neural differentiation. The immunoprecipitation experiments showed that RA enhanced the levels of PCAF, CBP, and P300 recruitment to RARα (Figure 6d). To further confirm the critical roles of RARs in regulating histone acetylation, RA signaling in SH-SY5Y cells was altered by the additional treatment with BMS195614 (RARα antagonist) and LE135 (RARβ antagonist). Reduced H3ac and H3K14ac levels were observed in the groups treated with RA and RAR antagonists compared with the RA-treated group (Figure 6e–g). In addition, after RAR antagonist treatment, mitochondrial function-related genes were downregulated (Figure 7a–d), and neurite outgrowth and neuronal markers were suppressed (Figure 7e–j). These results indicated that RA promoted HATs and increased histone acetylation via RA signaling, thereby regulating neural differentiation.

### 3.7. RIP140, a RAR Co-Repressor, Is Involved in Regulating Histone Acetylation during Neural Differentiation

**Figure 8:**
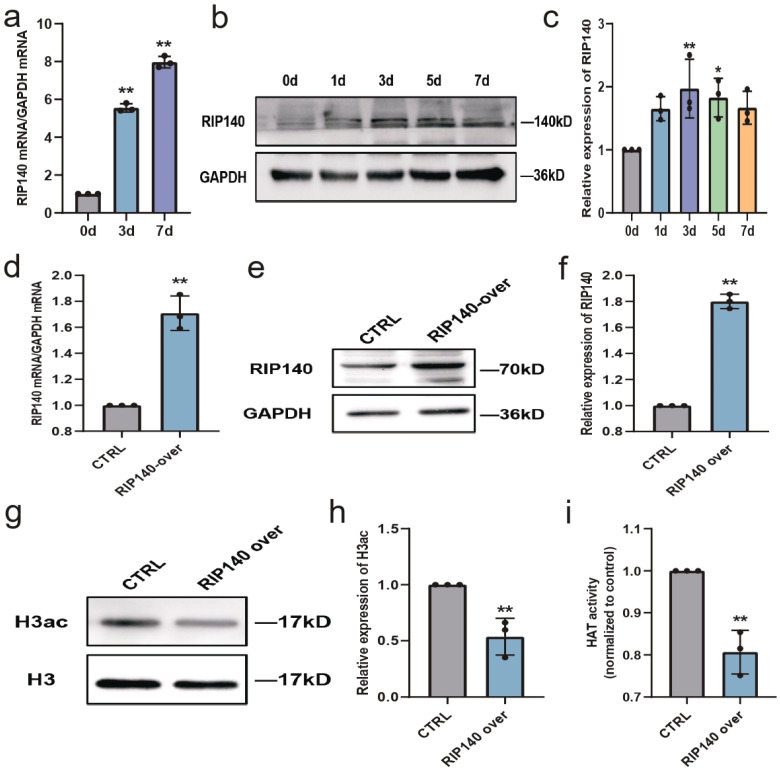
RIP140 is involved in regulating histone acetylation during neural differentiation. (**a**) RT-qPCR analysis of relative mRNA expression of *RIP140* in SH-SY5Y cells treated with RA for 3 and 7 days. *n* = 3; ** *p* < 0.01; one-way ANOVA. (**b**,**c**) Western blot analysis of relative protein expression of RIP140 in SH-SY5Y cells after RA treatment for 1, 3, 5, and 7 days. *n* = 3; * *p* < 0.05, ** *p* < 0.01; one-way ANOVA. (**d**–**f**) Relative mRNA and protein expression of RIP140 in RIP140-overexpressing N2a cells versus control N2a cells. *n* = 3; ** *p* < 0.01; *t*-test. (**g**,**h**) Western blot analysis of H3ac in control N2a cells and RIP140-overexpressing N2a cells treated with RA for 3 days. *n* = 3; ** *p* < 0.01; *t*-test. (**i**) Control N2a cells and RIP140-overexpressing N2a cells after RA treatment for 3 days were subjected to a HAT activity assay. *n* = 3; ** *p* < 0.01; *t*-test.

RIP140, a transcriptional target of RA, is a RAR co-repressor and induces self-limitation of RA signaling in the persistent presence of RA [27,28]. Reportedly, RIP140 can recruit HDACs to inhibit histone acetylation and silence genes [29,30]. Therefore, whether RIP140 mediates the self-limitation of RA signaling by regulating histone acetylation during neural differentiation was investigated. As shown in Figure 8, after RA treatment, both RIP140 mRNA and protein expression levels in SH-SY5Y cells were significantly upregulated (Figure 8a–c). Next, the stable RIP140-overexpressing N2a cell model (a *mouse* neuroblastoma cell line), which was established in our previous work, was used to investigate the relationship between RIP140 and histone acetylation. The RIP140-overexpressing system was verified at both the mRNA and protein levels (Figure 8d–f). After RA treatment for 3 days, H3ac in RIP140-overexpressing N2a cells was significantly decreased compared with that in control N2a cells (Figure 8g,h), and changes in HAT activity were consistent with the acetylation levels of histone H3 (Figure 8i).

**Figure 9:**
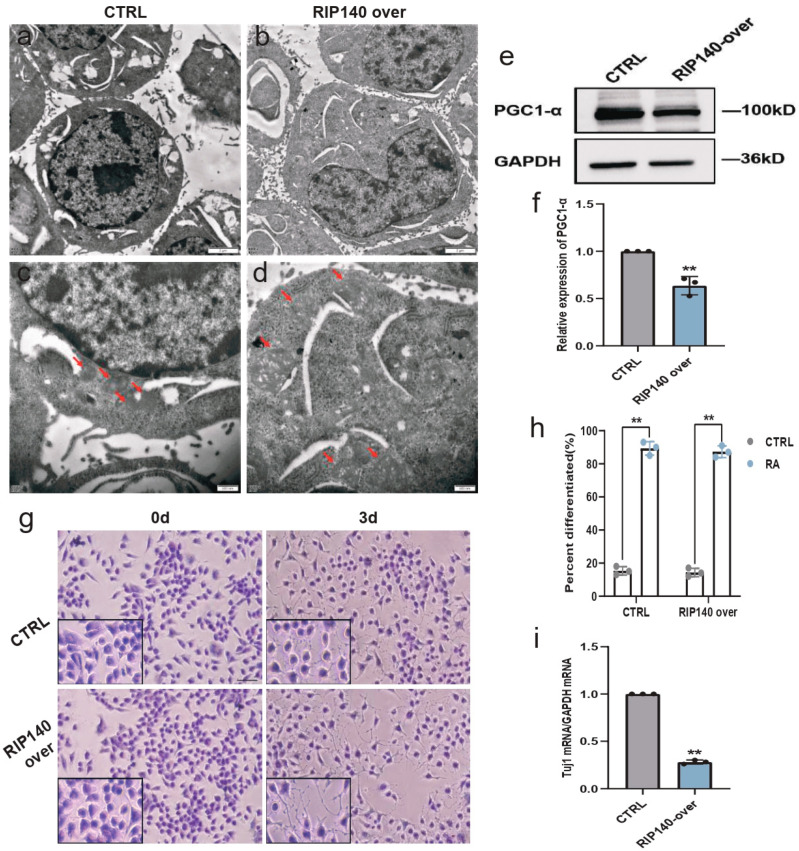
RIP140 overexpression downregulates the mitochondrial biogenesis marker PGC-1α and the neural differentiation marker *Tuj1*. (**a**–**d**) Ultrastructure of cells and mitochondria in control N2a cells and RIP140-overexpressing N2a cells treated with RA for 3 days. Mitochondria in control N2a cells were regular with clear lamellar cristae and mitochondria in RIP140-overexpressing N2a cells were swollen with disordered cristae. Red arrows show the mitochondria. Scale bars, 2 µm and 500 nm, respectively. (**e**,**f**) Western blot analysis of PGC-1α protein expression in control N2a cells and RIP140-overexpressing N2a cells after RA treatment for 3 days. *n* = 3; ** *p* < 0.01; *t*-test. (**g**,**h**) Morphology and differentiation rate of control N2a cells and RIP140-overexpressing N2a cells cultured in maintenance medium or stimulated with RA for 3 days. Three random fields in each group were selected to calculate the differentiation rate. Scale bar, 50 µm. Images in the black box show cells at a higher magnification. ** *p* < 0.01; *t*-test. (**i**) RT-qPCR analysis of *Tuj1* mRNA expression in control N2a cells and RIP140-overexpressing N2a cells after RA treatment for 3 days. *n* = 3; ** *p* < 0.01; *t*-test.

Next, whether RIP140 overexpression affects mitochondrial function and neural differentiation was investigated. TEM showed that the mitochondria were swollen and the mitochondrial crest was disordered in RIP140-overexpressing N2a cells, and the mitochondria were regular with a clear lamellar crest in control N2a cells (Figure 9a–d). Decreased expression of the mitochondrial biogenesis marker PGC-1α was also observed in the RIP140-overexpressing group (Figure 9e,f). Furthermore, H&E staining showed that both control cells and RIP140-overexpressing cells were almost differentiated after RA stimulation for 3 days, and the differentiation rate did not differ (Figure 9g,h). However, the expression of the neuronal marker *Tuj1* was significantly lower in RIP140-overexpressing cells than in control cells (Figure 9i), which indicated that *Tuj1* was inhibited in differentiated cells by RIP140-mediated negative feedback. All the above suggest that RIP140 is also involved in regulating histone acetylation during neural differentiation. RIP140 triggered negative feedback on RA target genes by inhibiting histone acetylation.

## 4. Discussion

**Figure 10:**
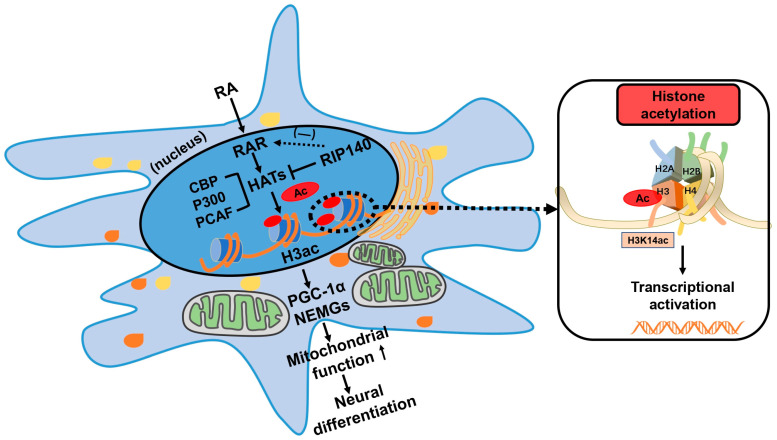
Working model of the epigenetic regulation of mitochondrial function during neural differentiation. RA activates HATs and enhances the acetylation of histone H3 by increasing the recruitment of HATs to RARs. The acetylation of histone H3 is involved in regulating mitochondrial function, thereby affecting neural differentiation. RIP140, a co-repressor of RAR, mediates functional negative feedback on RA signaling target genes.

In the present study, several key points regarding the epigenetic regulation of mitochondrial function during RA-induced neural differentiation were confirmed. First, RA significantly enhanced HAT activity and histone H3 acetylation. Second, histone H3 acetylation was involved in regulating mitochondrial function, thereby affecting neural differentiation. Third, RA promoted HAT-dependent histone acetylation via RA signaling by increasing the recruitment of HATs. Finally, during RA-induced neural differentiation, RIP140 was also involved in regulating histone acetylation and mediated functional negative feedback on target genes of RA signaling (Figure 10).

The critical role of chromatin dynamics in the regulation of gene expression during neural differentiation has been highlighted in numerous studies [5,20,31,32]. In addition, RA has been shown to be involved in regulating epigenetic changes and inducing enhanced histone acetylation in leukemia cells and in the rat hippocampus [21,33]. Here, we demonstrated that histone acetylation plays essential roles in RA-induced neural differentiation of SH-SY5Y cells. Histone acetylation occurs mainly on the tails of histone H3 or H4, and we selected acetylation of histone H3, which has been widely studied in neural differentiation. We detected the acetylation of several histone lysine residues (H3K9 and H3K14) that have been reported to be associated with transcriptional activation [34,35]. We found that RA particularly enhanced the acetylation of H3K14 and did not influence the acetylation of H3K9. Reportedly, H3K14ac is a necessary and sufficient condition for the decomposition of the promoter nucleosome and the transcriptional activation of genes, which is closely associated with neural differentiation [5,36,37]. In the present study, RA enhanced H3ac and H3K14ac during the neural differentiation of SH-SY5Y cells. The upregulated expression and activity of HATs (CBP, P300, PCAF), which directly catalyze histone acetylation, caused RA-induced increased histone acetylation. Furthermore, neural differentiation was blocked when histone acetylation was inhibited by a HAT inhibitor, indicating that histone acetylation is important for RA to maintain neural differentiation. Several studies have shown that epigenetically active compounds, such as the HDAC inhibitors Trichostatin A (TSA) and sodium butyrate (NaBt), can improve the differentiation potential of RA-treated SH-SY5Y cells, which provided a valuable insight into the regulation of histone acetylation to promote neural differentiation [38,39].

Mitochondria have been shown to be central regulators of neural stem cell fate decisions by generating energy, regulating subcellular Ca^2+^ levels, and maintaining redox homeostasis [13]. Mitochondria are important for promoting neural differentiation, which, in turn, contributes to neurodevelopment and cognitive processes [4,14,40,41]. In our previous study, mitochondrial function was increased during the neural differentiation of SH-SY5Y cells [10]. Here, we showed that RA induced mitochondrial maturation and morphological changes, and upregulated mitochondrial function-related genes; these effects were all blocked after inhibiting histone acetylation with C646 treatment. These results indicate that histone acetylation is involved in regulating mitochondrial function, thereby affecting neural differentiation. Martine et al. revealed that the HDAC inhibitor NaBt improves mitochondrial biogenesis and oxidative metabolism, leading to enhanced neural progenitor cells, which also highlighted the critical role of histone acetylation in mitochondrial function during neural differentiation [4].

RA performs most of its physiological functions through RA signaling. RARs regulate the expression of downstream genes by binding to and then activating the promoter elements of target genes. Co-transcriptional complexes possessing HAT or HDAC activity are recruited after RAR activation, which remodel the chromatin and regulate gene transcription and expression [22,42,43]. The present study results showed that RA enhanced HAT (CBP, P300, PCAF) recruitment to RARs during neural differentiation. Blocking RA signaling pathways with RAR antagonists inhibited histone acetylation and subsequently downregulated mitochondrial function and blocked neural differentiation. Therefore, the RA-induced increase in histone acetylation during neural differentiation was mediated by RA signaling.

*RIP140* is located on human chromosome 21 and is closely associated with the pathogenesis of DS [44]. RIP140 overexpression has been associated with mitochondrial dysfunction and impaired neural differentiation [23]. In previous studies, RIP140 was shown to be a co-repressor of nuclear receptors through directly recruiting HDACs to silence genes [29]. RIP140, a transcriptional target of RA, induces a functional negative feedback that limits the continued activation of RARs, thus maintaining the homeostasis of hormonal signaling [27,45]. In the present study, whether RIP140 induced self-limitation of RA signaling by inhibiting histone acetylation was investigated. Due to the difficulty and instability of constructing a gene overexpression system using SH-SY5Y cells, the stable RIP140-overexpressing N2a cell line established in our previous work was used to detect the effect of RIP140 on histone acetylation. As expected, RIP140 overexpression repressed histone acetylation, and the mitochondrial biogenesis marker PGC-1α and neuronal marker Tuj1 were subsequently downregulated during RA-induced neural differentiation. Previous studies have reported that PGC-1α is negatively controlled by the co-repressor RIP140, which has been called the RIP140/PGC-1α axis [23]. In Figure 3c, we found that PGC-1α initially increased and then decreased by 7 days, which also confirmed the existence of the RIP140/PGC-1α axis. It suggested that RA induced the upregulation of RIP140 during neural differentiation. As RIP140 constantly increased, it limited continued expression of target genes of RAR by repressing histone acetylation and thereby mediating negative feedback on RA. Given our findings of a direct link between RIP140 and histone acetylation during neural differentiation, the role of RIP140 cannot be ignored in the development of therapies for neurodevelopmental disorders.

In conclusion, the study results contribute to a better understanding of the epigenetic regulation of mitochondrial function during RA-induced neural differentiation and indicate that RA functions as a modulator of the histone acetylation-dependent gene expression network. Furthermore, genetic changes are difficult to reverse, and epigenetic aberrations can be pharmaceutically reversible. Thus, the enhanced HATs and the acetylation of their downstream substrates are promising therapeutic strategies targeting specific epigenetic mechanisms for neurodevelopmental disorders.

There were several limitations in our study. First, only one cell line model was used, and the results should be further confirmed in the differentiation of neurospheres or NSCs, which would be more relevant and significant. Second, there are limitations in the detection of mitochondrial function in our study, since we only evaluated morphology, biogenesis markers, and electron transcription chain activity. More assays, including those for mitochondrial membrane potential, oxidative metabolism, and ATP production, will need to be performed in the future. Third, the present study is largely correlative and more specific mechanisms are required, such as the epigenetic regulation of transcriptional factors related to neural differentiation. Previous studies have demonstrated that increased H3K14ac altered the chromatin state of the promoters of *Ngn1* and *NeuroD1* and activated their transcription; then, *Ngn1* and *NeuroD1* initiated and sustained the commitment to neuronal fates during melatonin-induced neural differentiation of NSCs [5]. More H3ac/H3K14ac ChIP studies for promoters of key transcriptional factors should be conducted in the future.

## Figures and Tables

**Table 1:** RT-qPCR primers.

Genes	Forward (5′-3′)	Reverse (5′-3′)
*TUJ1*	CAAGGTGCGTGAGGAGTAT	GTCTGACACCTTGGGTGAGG
*Tuj1* (M)	ACCCCGTGGGCTCAAAAT	CCGGAACATGGCTGTGAACT
*MAP2*	CTCAGCACCGCTAACAGAGG	CATTGGCGCTTCGGACAAG
*PGC1-α*	TGGTGCCACCACCATCAAAGA	TCACCAAACAGCCGCAGACTG
*NDUFS3F*	ATCATATGGCGGCGGCGGC	TGCTCGAGCTACTTGGCATCAGGCTTC
*ANT1*	CTCTCCTTCTGGAGGGGTAAC	GAACTGCTTATGCCGATCCAC
*ANT2*	GGGTCAAGCTGCTGCTGCAGG	CGGAATTCCCTTTCAGCTCCAGC
*ANT3*	CACCAAGTCCGACGGCATCCG	ACGGTTGAGGATTCTACGTGG
*RIP140*	CCCATTTGCAGCAGTATTCTC	GTAACTGCCAACATCCTTCTG
*Rip140* (M)	GAACCTGGGCTTTTGAATGG	GTTTTGGTCAGTCTTGGAGAGTCTT
*RARα*	CTCTCCACCAAGTGCATCATTAAG	CAGCCTTGAGGAGGGTGATC
*RARβ*	TGACAGCTGAGTTGGACGAT	AGCACTGGAATTCGTGGTGT
*RARγ*	CTGTGCGAAATGACCGGAAC	CTGCACTGGAGTTCGTGGTA
*GAPDH*	GTGAAGCAGGCGTCGGAG	GCGTCAAAGGTGGAGGAGTG
*β-actin* (M)	CTGTCCCTGTATGCCTCTG	ATGTCACGCACGATTTCC

## Data Availability

The data presented in this study are available on request from the corresponding author.
